# Overexpression of SIRT6 in the hippocampal CA1 impairs the formation of long-term contextual fear memory

**DOI:** 10.1038/srep18982

**Published:** 2016-01-06

**Authors:** Xi Yin, Yuan Gao, Hai-Shui Shi, Li Song, Jie-Chao Wang, Juan Shao, Xu-Hong Geng, Gai Xue, Jian-Li Li, Yan-Ning Hou

**Affiliations:** 1 Department of Functional region of Diagnosis, Hebei Medical University Fourth Hospital, Hebei Medical University, Shijiazhuang 050011, China; 2 Department of Biochemistry and Molecular Biology, College of basic medicine, Hebei Medical University, Shijiazhuang 050017, China; 3 Department of vasculocardiology, Hebei Province Geriatric Hospital, Shijiazhuang, 050011, China; 4 Department of Senile Disease, the Third Hospital of Hebei Medical University, Shijiazhuang 050000, China; 5 Department of Pharmacy, the Bethune International Peace Hospital of PLA, Shijiazhuang 050082, China

## Abstract

Histone modifications have been implicated in learning and memory. Our previous transcriptome data showed that expression of sirtuins 6 (SIRT6), a member of Histone deacetylases (HDACs) family in the hippocampal cornu ammonis 1 (CA1) was decreased after contextual fear conditioning. However, the role of SIRT6 in the formation of memory is still elusive. In the present study, we found that contextual fear conditioning inhibited translational expression of SIRT6 in the CA1. Microinfusion of lentiviral vector-expressing SIRT6 into theCA1 region selectively enhanced the expression of SIRT6 and impaired the formation of long-term contextual fear memory without affecting short-term fear memory. The overexpression of SIRT6 in the CA1 had no effect on anxiety-like behaviors or locomotor activity. Also, we also found that SIRT6 overexpression significantly inhibited the expression of insulin-like factor 2 (IGF2) and amounts of proteins and/or phosphoproteins (e.g. Akt, pAkt, mTOR and p-mTOR) related to the IGF2 signal pathway in the CA1. These results demonstrate that the overexpression of SIRT6 in the CA1 impaired the formation of long-term fear memory, and SIRT6 in the CA1 may negatively modulate the formation of contextual fear memory via inhibiting the IGF signaling pathway.

The precise molecular mechanism underlying memory formation has been studied intensely for centuries and is still far from being understood. Deeper understanding of the neurobiological mechanisms of memory function has significant clinical implications and may aid in the development of more effective therapies for amnesia, cognitive decline, and fear and anxiety disorders^1^,^2^,^3^. In the laboratory, the mechanisms that underlie fear memory are often studied using Pavlovian fear conditioning (FC) paradigms. In the contextual version of this paradigm, a single exposure of rodents to an originally neutral conditioned stimulus (CS; e.g., context) followed by an aversive unconditioned stimulus (US; e.g., an electric foot-shock) elicits the acquisition of conditioned fear. On the basis of associative learning, the animals display an inborn aversive freezing behavior upon re-exposure to the conditioned context. A growing body of evidence demonstrates that the hippocampus plays a central role in modulating the formation, expression, and extinction of fear following this form of contextual FC^4^,^5^,^6^,^7^,^8^. For example, bilateral injection of insulin-like growth factor 2 (IGF2) into the dorsal hippocampus of rats or mice enhances fear memories and facilitates fear extinction^7^,^9^. Increasing evidence emphasizes a unique function in memory for each hippocampal subregion, with the CA3 area acting as an auto-associative memory network, the CA2 region is essential for social memory and the CA1 area is rather involved in the consolidation process of contextual memory^10^,^11^,^12^.

The Histone deacetylases (HDACs) family is comprised of Class I (HDAC1, 2, 3 and 8), class II a (HDAC, 5, 7 and 9), II b (HDAC 6 and 10), III (the sirtuins) and class IV (HDAC 11) enzymes^13^. Growing evidence indicates that HDACs are powerful modulators of long-term potentiation (LTP) as well as memory formation^14^,^15^,^16^. Increased histone-tail acetylation facilitates various types of long-term memories, including hippocampus-dependent contextual fear conditioning and spatial memory^17^,^18^,^19^,^20^, amygdala-dependent auditory fear conditioning and amygdala- and nucleus accumbens (NAc)-dependent drug-associated memories^21^,^22^. Pre-training administration inhibitors of HDAC [trichostatin A (TSA) or sodium butyrate (NaB)] enhanced induction of long term potentiation (LTP) in area CA1^20^.

Sirtuins (SIRTs), mainly include seven members, SIRT1-7 in mammals, possess a highly conserved central NAD^+^ binding site and common catalytic domain^23^. SIRT1 has been the most extensively studied, and accumulating evidence suggests that SIRT1 plays a protective role in normal brain physiology and neurological disorders. SIRT1 knockout mice exerted cognitive deficits and exhibited a decrease in dendritic branching, branch length and complexity of neuronal dendritic arbors, and show altered hippocampal gene expression, which plays important roles in synaptic and structural functions^24^,^25^. Hippocampal SIRT2 is involved in the modulation of depressant-like behaviors^26^. SIRT6 has both nuclear ADP-ribosyltransferase activity and deacetyltransferase activity, and is considered to have a leading role in regulating genomic stability, cellular metabolism, stress response, aging, cancer, obesity and cardiac hypertrophy^27^,^28^,^29^,^30^,^31^,^32^,^33^,^34^,^35^,^36^. SIRT6 negatively controls insulin-like factor (IGF)/Akt signaling at the level of chromatin and culminates in the development of cardiac hypertrophy and heart failure in mice. Male transgenic mice overexpressing Sirt6 displayed lower serum levels of insulin-like growth factor 1 (IGF1) and had a significantly longer lifespan than wild-type mice^34^,^37^. Also, SIRT6 attenuates NF-κB signaling via H3K9 deacetylation at chromatin and prevents premature and normal aging^38^. However, the role of SIRT6 in the formation of contextual fear memory is unknown. In the present study, using a lentivirus-mediated SIRT6 overexpression procedure, we investigated the effect of SIRT6 in the CA1 region on the formation of contextual fear memory and the underlying mechanism.

## Materials and Methods

### Subjects

Male Sprague Dawley rats (3 months old, weighing 240–260 g on arrival) were obtained from the Laboratory Animal Center, Peking University Health Science Center and were housed in groups of five under controlled temperature (23 ± 2 °C) and humidity (50 ± 5%), and maintained on a 12-h light/dark cycle with access to food and water *ad libitum*. All of the experiments were performed according to the National Institutes of Health Guide for the Care and Use of Laboratory Animals and all of the experimental protocols were approved by the Biomedical Ethics Committee for animal use and protection of Hebei Medical University. The behavioral experiments were conducted during the dark phase of the cycle.

### Brain sample preparation and microarray analysis

The rats were decapitated, and brains were extracted based on our previous study. The rats were killed by rapid decapitation without anesthesia 1.5 h after the end of fear conditioning. The hippocampal CA1 region was removed from the brain, snap-frozen in liquid nitrogen, and stored at −80 °C until analysis. Total RNA of CA1 tissues was isolated using the RNApre Pure Tissue kit (TIANGEN BIOTECH CO., LTD Beijing China) according to manufacturer’s instructions, including a DNase digestion step. After having passed RNA measurement on the NanoDrop ND-1000 (NanoDrop Technologies, Wilmington, DE, USA) by measuring absorbance (A280, A260, A230), RNA concentrations and quantity, and then denaturing gel electrophoresis. For microarray analysis, Agilent Array platform (Shanghai KANGCHEN, Shanghai China) was employed. The sample preparation and microarray hybridization were performed based on the manufacturer’s standard protocols. Briefly, total RNA from each sample was amplified and transcribed into fluorescent cRNA with using the manufacturer’s Agilent’s Quick Amp Labeling protocol (version 5.7, Agilent Technologies). The labeled cRNAs were hybridized onto the Rat LncRNA Array (4 × 44 K, Arraystar, KangChen Bio-tech Inc. Shanghai China). After having washed the slides, the arrays were scanned by the Agilent Scanner G2505C.

### Design, construction, and validation of lentiviral vectors for SIRT6 overexpression

The construction and use of the self-complementing lentiviral vectors were based on our previous studies with minor modifications^39^,^40^. pGV287 vector plasmids, containing the enhanced green fluorescence protein (eGFP) coding sequence (obtained from Shanghai Genechem, Shanghai, China) were constructed for the production of lentiviruses mediated SIRT6 expression. SIRT6 was amplified by polymerase chain reaction (PCR) and subcloned into the GV287 vector using *BamH* I and *Age* I restriction sites. All of recombined vectors (pGV287-SIRT6) were then transfected into human embryonic kidney 293 cells. Approximately 48 h post-transfection, cells were harvested, purified by centrifugation, and stored at −80 °C.

### Surgery

The rats were anesthetized with sodium pentobarbital (50 mg/kg, i.p.) and then placed in a stereotaxic apparatus. Identical stainless-steel guide cannulae (22 gauge) were bilaterally implanted into the CA1 [anterior/posterior (AP), −3.8 mm; lateral (L), ±2.0 mm; dorsal/ventral (DV), −2.5 mm] or dentate gyrus (DG) [AP − 3.8 mm, L ± 2.0 mm, DV − 3.1 mm]^41^,^42^. After surgery, the rats were allowed 7 days to recover, during which time they were handled until the start of training.

### Intracranial Injections of Lentiviruses

The experimental parameters that were used for the virus injections were based on our and others previous work^39^,^40^. The LV_SIRT6-GFP_ and LV_GFP_ lentiviruses [(1 × 10^9^ viral genomes, dissolved in phosphate-buffered saline (PBS)] were injected into the CA1 with 10 μl Hamilton syringes that were connected via polyethylene-50 tubing to 30-gauge injectors. The Hamilton syringes were connected to an infusion pump (RWD Life Science CO., LTD, China). The viruses were delivered bilaterally over 10 min at an infusion rate of 0.1 μl/min (total volume, 1 μl per side), and the injectors were left in place for an additional 5 min to allow diffusion before removing them. At the end of the experiments, the rats were anesthetized with sodium pentobarbital (100 mg/kg, i.p.) and transcardially perfused. Cannula placements were assessed using Nissl staining with a section thickness of 40 μm under light microscopy.

### Contextual Fear Memory

Contextual fear conditioning was conducted in four identical isolated shock chambers (Beijing Macro Ambition S&T Development Co., Ltd, Beijing, China). The contextual fear conditioning procedure was modified from previous studies^43^,^44^. The rats were handled for 3 days before conditioning. On the day of the experiments, they were placed into the conditioning chamber and allowed to explore the chamber for 2 min, after which they received an electric footshock (0.8 mA, 1 s). The 2 min/1 s procedure was repeated a total of three times, and the rats were allowed to explore the conditioning chamber for an additional 1 min, and then were returned to their homecages and left undisturbed in the testing room. All chambers were cleaned with 75% alcohol to eliminate any residual odor. The short-term memory (STM) test was performed 1.5 h later by re-exposing the rats for 5 min into the conditioning context. Freezing, defined as a lack of movement except for heart beat and respiration associated with a crouching posture, was recorded and analyzed using an animal behavior video analysis system (Beijing Macro Ambition S&T Development, Beijing, China). For long-term memory (LTM) test, the memory test was performed 24 hrs and 7 ds after the foot shock training. Behavioral score was assessed by measuring the percentage of time spent freezing during the 5-min test period.

### Locomotor Activity (LA) Test

Locomotor activity was measured according to our previous studies using an automated video tracking system (DigBehv-LM4, Shanghai Jiliang Software Technology Co. Ltd, Shanghai, China)^44^,^45^. A monochrome video camera was mounted on top of each chamber. All of the chambers were connected to a computer that recorded locomotion. The video files (stored on the computer) were analyzed using DigBehv analysis software. Animals were transported to the experimental room (luminosity at the level of the LA was 30 W) an hour before starting the experiment and were left undisturbed. In brief, the rats were placed into the chamber and allowed to explore the chamber for 5 min. Locomotor activity is expressed as the total distance traveled during the 5 min test and the duration time in the central zone were also recorded to reflect anxiety-like behavior.

### Elevated Plus Maze (EPM) test

Animals were transported to the experimental room (luminosity at the level of the EPM was 30 W) an hour before starting the experiment and were left undisturbed. The EPM test was based on our previous studies^40^,^46^. Briefly, each rat was first placed in the central zone of the EPM. The rat was allowed to freely explore the maze for 5 min under dimillumination. The number of entries into and time (in seconds) spent in the open/closed arms were recorded by two independent observers who were blind to the group assignments and sat quietly at a distance of 2.5 m from the maze.

### Western Blot Assays

The Western blot assays were based on our previous studies^39^,^47^. All of the rats were decapitated, and the brains were rapidly extracted, frozen and stored in a −80 °C freezer. Afterward, bilateral tissue punches (16 gauge) of the CA1 area were placed in a 1.5 ml microtube that contained ice-cold 100 μl of homogenization buffer (Beyotime Biotechnology, China) for 30 min. The tissue samples were then homogenized (10–15 s three times at 5 s intervals) with an electrical disperser (Wiggenhauser, SdnBhd), the homogenates were centrifuged at 1000 × g for 10 min at 4 °C. The supernatant was used for subsequent Western blot. The protein concentrations of all of the samples were determined using the bicinchoninic acid assay (Beyotime Biotechnology, China). The samples were further diluted in RIPA lysis buffer to equalize the protein concentrations. Four × loading buffer [16% glycerol, 20% mercaptoethanol, 2% sodium dodecyl sulfate (SDS), and 0.05% bromophenol blue] was added to each sample (3:1, sample: loading buffer) before boiling for 3 min. The samples were cooled and subjected to SDS-polyacrylamide gel electrophoresis (10% acrylamide/0.27% *N*,*N*′-methylenebisacrylamide resolving gel) for approximately 40 min at 80 V in stacking gel and approximately 1 h at 120 V in resolving gel. Proteins were electrophoretically transferred to polyvinylidene fluoride transfer membranes (Millipore, Bedford, MA, USA) at 250 mA for 2.5 h. Membranes were washed with TBST (Tris-Buffered Saline plus 0.05% Tween-20, pH 7.4) and then dipped in blocking buffer (5% bovine serum albumin [BSA] in TBST) overnight at 4 °C. The next day, the membranes were incubated for 1 h at room temperature on an orbital shaker with anti-SIRT6, anti-SIRT1, anti-SIRT2 (1:1000; Sigma Aldrich, USA), anti-IGF1, anti-IGF2, anti-p-Akt, anti-Akt, anti-mammalian target of rapamycin (anti-mTOR), anti-p-mTOR, or anti-β-actin antibody (1:2000; Santa Cruz Biotechnology, Santa Cruz, CA, USA) in TBST plus 5% BSA. After three 5-min washes in TBST buffer, the blots were incubated for 45 min at room temperature on a shaker with horseradish peroxidase-conjugated secondary antibody (goat anti-mouse IgG forβ-actin and goat anti-rabbit for others; Santa Cruz Biotechnology, Santa Cruz, CA, USA) diluted 1: 5000 in blocking buffer. The blots were then washed three times for 5 min each in TBST and incubated with a layer of Super Signal Enhanced chemiluminescence substrate (Detection Reagents 1 and 2, 1:1 ratio, Applygen Technologies, Beijing, China). Excess mixture was dripped off before the blots were wrapped with a clean piece of plastic wrap (no bubbles between blot and wrap), and the blots were exposed to X-ray film (Eastman Kodak Company) for 5–60 s. Band intensities were quantified using Quantity One software (version 4.4.0, Bio-Rad, Hercules, CA, USA).

### Immunohistochemistry

Immunofluorescence was based on our previous studies^40^,^48^. After the behavioral experiments, the rats were anesthetized and perfused with 0.01 mol/L phosphate-buffered saline (PBS) and 4% paraformaldehyde, pH 7.4. The brains were then extracted and removed in 4% paraformaldehyde for 24 h. Subsequently, the brains were placed in 30% sucrose for approximately 24–48 h, frozen, coronally sectioned at 10 μm using a sliding microtome, and stored in PBS. Brain slices were then mounted on Super frost/plus slides, and eGFP expression was screened using a fluorescent microscope at the injection sites. Representative images were captured at the same time.

### Statistical Analysis

The data expressed as mean ± SEM. The statistical analysis was performed with SPSS software. Analysis of variance (ANOVA) with appropriate between- and/or within-subjects factors for each experiment (see Results). Significant main effects and interactions (*p* < 0.05, two-tailed) in the factorial ANOVAs were followed by one-way/two-way ANOVAs and the Least Significant Difference *post hoc* test. Effect size (ES) was also reported [ES = sqrt (F/n)].

## Results

### Experiment 1: Contextual fear conditioning induced decreased SIRT6 expression of SIRT6 in the CA1

**Figure 1:**
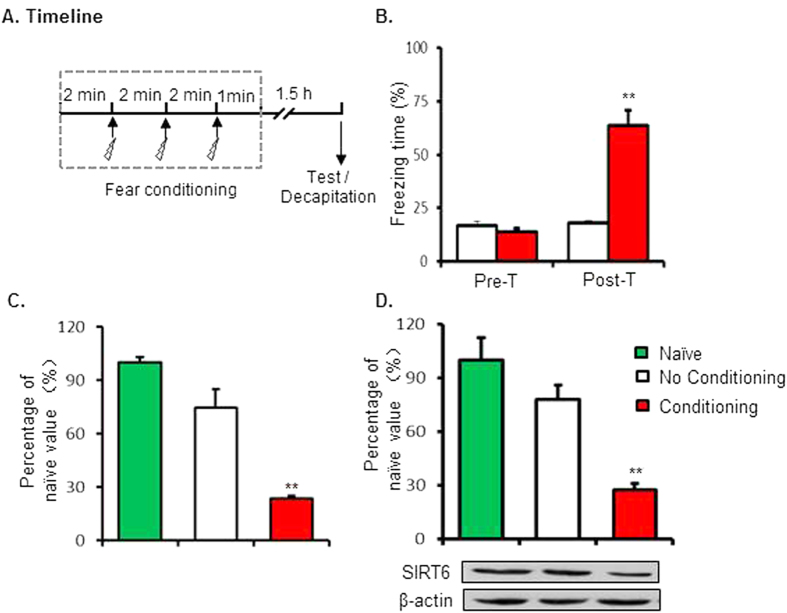
Decreased SIRT6 expression in the CA1 after contextual fear conditioning. (**A**) Timeline of the experiment. (**B**) Rats in fear conditioning group exhibited higher freezing behaviors. Microarray analysis (**C**) and Western blot analysis (**D**) of SIRT6 in CA1 tissues lysates of rats 1 h after fear conditioning. **p < 0.01 compared with “No Conditioning” group.

In experiment 1, we tested the effects of fear conditioning on protein of SIRT6. Two groups of rats (n = 12–14 per group) underwent the contextual fear conditioning, and then were tested for STM 1.5 h after fear conditioning. Rats were decapitated immediately after the STM test and tissue of CA1 region was isolated for microarray analysis (n = 3–4 per group) and western blot analysis (n = 3–5 per group) separately (Fig. 1A). The freezing scores (Pre-con: 16.8 ± 1.7%, 13.8 + 1.5%; Post-Con: 18.0 ± 1.2%, 63.6 + 6.9% for No C and C group separately) were analyzed using two-way ANOVA. Using the between-subjects factor of fear conditioning (No conditioning and Conditioning) and the within-subjects factor of test condition (Pre-test and test) This analysis revealed significant effects of fear conditioning (F_1,14_ = 24.592, p < 0.001 ES = 0.973), test condition (F_1,14_ = 70.289, p < 0.001, ES = 1.644) and the interaction of fear conditioning X test condition (*F*1,14 = 60.081, *p* < 0.001,ES = 1.520; Fig. 1B). The post hoc analysis showed that rats exhibited increased fear response after fear conditioning. One-way ANOVA analysis of the data showed that both mRNA level (100 ± 3.0, 74.3 ± 10.5, 23.5 ± 1.4 separately) and protein level (100 ± 12.7, 78.1 ± 8.1, 27.8 ± 3.4 separately) of SIRT6 in the CA1 region were decreased after fear conditioning (F_2,8_ = 16.574, p < 0.0001, ES = 1.051; F_2,14_ = 17.168, p < 0.0001, ES = 1.070; Fig. 1C,D and Fig. S1).

### Experiment 2: Intra-CA1 injection of LVSIRT6-GFP increased the SIRT6 protein levels

**Figure 2:**
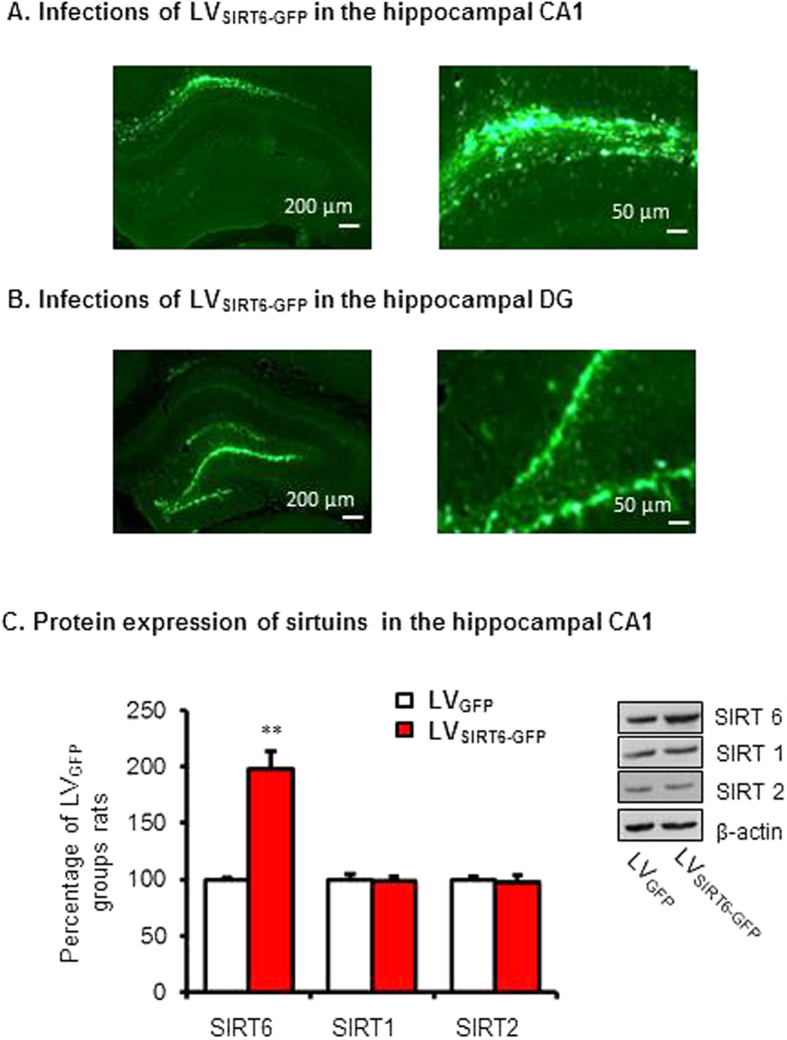
Lentiviral SIRT6-GFP infection in the CA1 enhanced the expression of SIRT6 in the CA1 of the hippocampus. Lentivirus-mediated GFP expression in the CA1 (**A**) or DG (**B**). (**C**)Western blot analysis of SIRT6 in the CA1 after LV_SIRT6-GFP_ infusion compared with infusion of LV_GFP_ alone. No significant differences in other isoforms were found in the CA1. **p < 0.01.

For further investigate the function of SIRT6 in memory formation, we constructed and microinjected LV_SIRT6-GFP_ into the CA1 and examined whether LV_SIRT6-GFP_ specifically increased the protein expression of SIRT6. Two groups of rats (*n* = 8 per group) received microinjections of LV_SIRT6 -GFP_ or LV_GFP_. Ten days later, all of the rats were euthanized to detect SIRT6, SIRT1 and SIRT2 protein levels in the CA1. Figure 2A showed the expression of EGFP in the CA1 of rats. The analysis of the Western blot data using one-way ANOVA revealed that SIRT6 expression in the CA1 was significantly increased 10 days after lentivirus infection in the group infused with LVSIRT6-GFP (198.2 ± 15.1%) compared with the group infused with LVGFP (100 ± 2.1%) in the CA1 (*F*1,9 = 41.412, *p* < 0.0001, ES = 2.035; Fig. 2C and Fig. S2). No significant differences were found in the protein levels of SIRT1 or SIRT2 in the CA1 region (all *p* > 0.05; Fig. 2C).

### Experiment 3: Effects of SIRT6 overexpression in the CA1 on short-term and long-term memory after contextual fear conditioning

**Figure 3:**
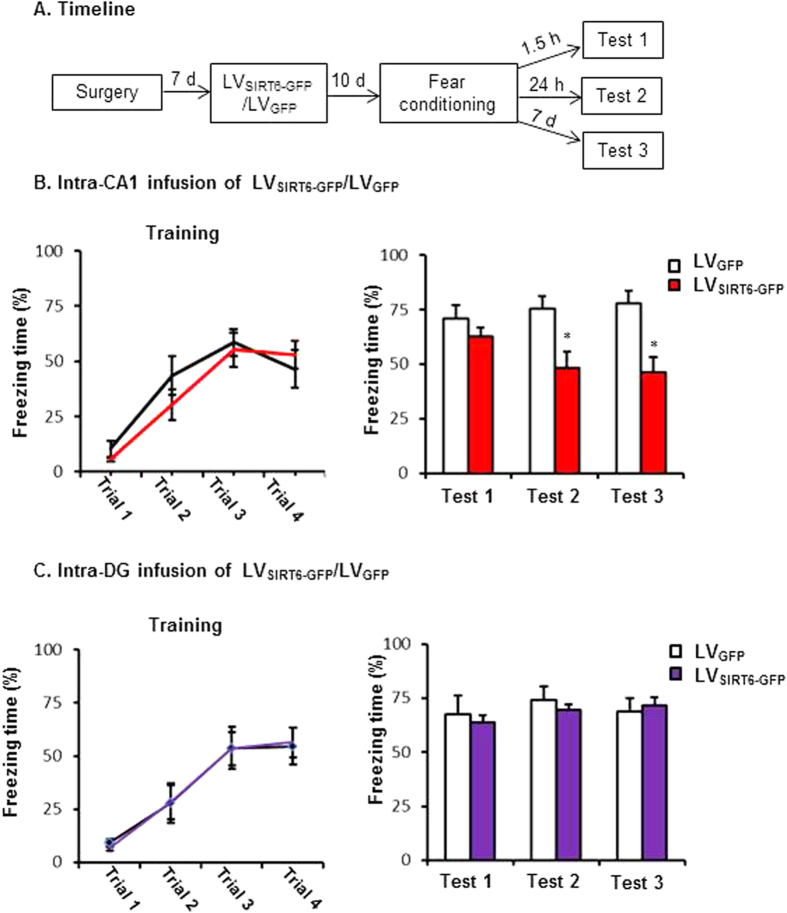
SIRT6 overexpression in the CA1 impaired the long-term fear memory formation. (**A**) Timeline of the experiment. 10 days after LV_SIRT6-GFP_ or LV_GFP_ infusions, all of the rats underwent fear conditioning and were tested for the fear response at 1.5 h, 24 h and 7 days after fear conditioning. (**B**) Rats received intra-CA1 infusion of LV_SIRT6-GFP_ exhibited decreased fear response at 24 h, 7 day but not during the fear conditioning process or 1.5 h post-conditioning compared with the LV_GFP_ infusion group. (**C**) Rats received intra-DG infusion of LV_SIRT6-GFP_ had no significant effects on fear response during fear conditioning process, or 1.5 h, 24 h, 7 d after fear conditioning compared with the LV_GFP_ group. *p < 0.05. The behavioral data are expressed as mean ± SEM.

We then assessed whether SIRT6 overexpression in the CA1 enhances the formation of fear memory. Two groups of rats (*n* = 11–12 per group) received LV_SIRT6-GFP_ or LV_GFP_ infusions into the CA1 region. Ten days later, the rats underwent contextual fear conditioning and were tested 1.5 h (short-term memory), 24 hs and 7 days (long-term memory) later (Fig. 3A). The freezing scores during fear conditioning and at 1.5 h (71.1 ± 8.4% and 62.7 ± 4.0%), 24 hrs (75.5 ± 7.0% and 48.3 ± 7.5%) and 7 days (77.8 ± 5.9 and 46.1 ± 7.3 &) after fear conditioning were analyzed using one-way ANOVA (LV_SIRT6-GFP_ and LV_GFP_). This analysis revealed a significant effect of lentivirus type in the CA1 24 h and 7 day after fear conditioning (*F*1,21 = 6.670, *p* = 0.017, ES = 0.667;*F*1,21 = 10.646, *p* = 0.004, ES = 0.822; Fig. 3B) but not during fear conditioning period and 1.5 h later after fear conditioning (all p > 0.05, Fig. 3B). These data indicate that the overexpression of SIRT6 in the CA1 had no effect on short-term fear memory but impaired the formation of long-term fear memory.

To exclude the region-specific effects of SIRT6 on the formation of contextual fear memory, Two groups of rats (n = 7–8 per group) received LV_SIRT6-GFP_ or LV_GFP_ infusions into the DG region. Figure 2B showed the expression of EGFP in the DG of rats. The rats underwent contextual fear conditioning and were tested 1.5 h (short-term memory), 24 hrs and 7 days (long-term memory) later. This analysis revealed no significant effects of lentivirus type in the DG region during fear conditioning, or 1.5 h, 24 h and 7 day after fear conditioning (all p > 0.05 Fig. 3C).

### Experiment 4: Effects of SIRT6 overexpression in the CA1 on the maintenance and expression of long-term contextual fear memory

**Figure 4:**
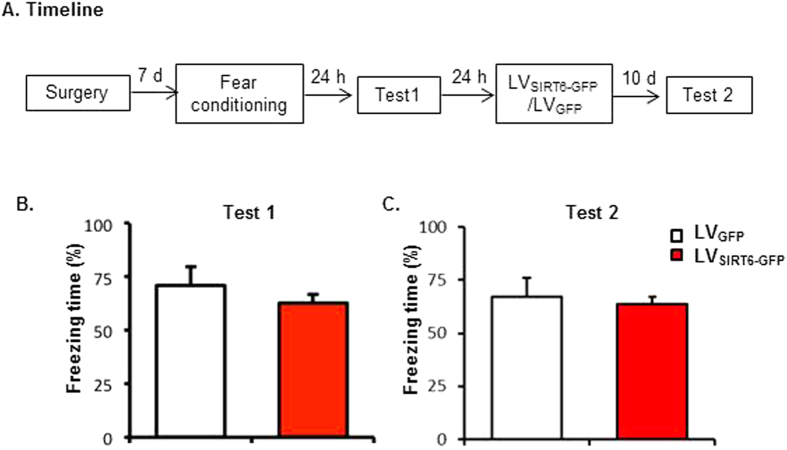
SIRT6 overexpression in the CA1 had no effect on maintenance and expression of contextual fear memory. (**A**) Timeline of the experiment. (**B**) post-conditioning intra-CA1 infusion of LV_SIRT6-GFP_ had no effects on long term memory formation and the following expression of fear response.

To confirm SIRT6 overexpression interfere with the formation rather than its maintenance and/or expression of long-term contextual fear memory, two groups of rats (n=8 per group) were trained fear conditioning, after a 24-h test, rats received LV_SIRT6-GFP_ or LV_GFP_ infusions into the CA1 region, ten days later, both groups of rats were tested (Fig. 4A). The results revealed that all rats formed long-term memory 24 hrs after contextual fear conditioning (Fig. 4B) and intra-CA1 infusion of LVSIRT6-GFP after fear conditioning had no significant effects on the expression and maintenance of contextual fear memory (Fig. 4C).

### Experiment 5: Effect of SIRT6 overexpression in the CA1 on locomotor activity (LA) and anxiety-like behaviors

**Figure 5:**
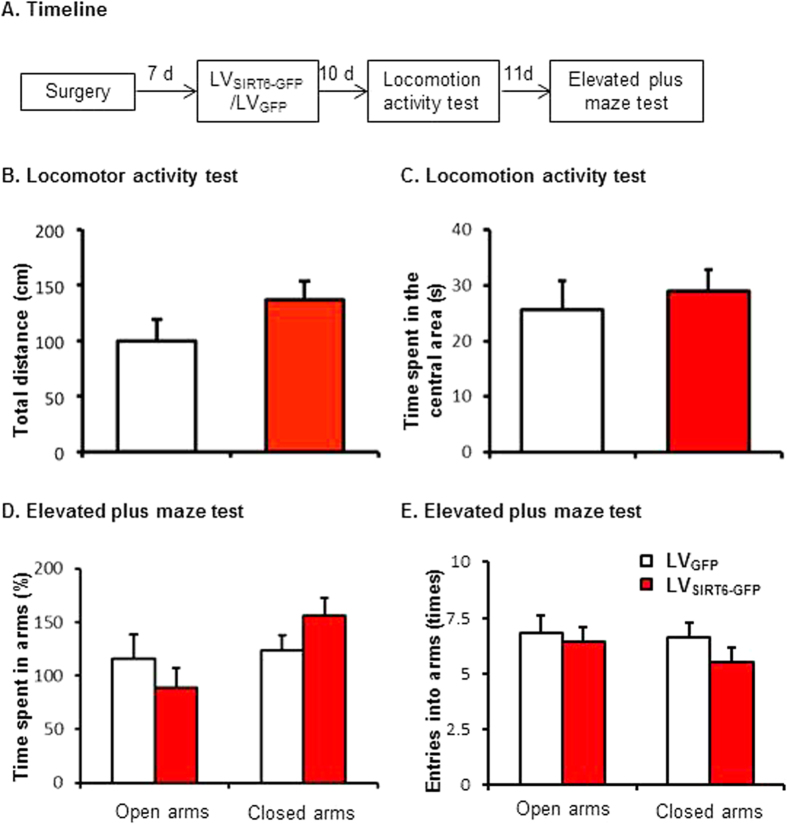
SIRT6 overexpression in the CA1 had no effect on locomotor activity or anxiety-like behavior. (**A**) Timeline of the experiment. 10 days after LV_SIRT6-GFP_ or LV_GFP_ infusions, all of the rats underwent the locomotor activity and elevated plus maze tests. Rats received intra-CA1 infusion of LV_SIRT6-GFP_ or LV_GFP_ exhibited similar total distance (**B**) and time spent in the central area (**C**) in the locomotor activity test and similar time spent in open/closed arms (**D**) and entries into open/closed arms (**E**) in the elevated plus maze test.

To exclude the possibility that SIRT6 overexpression in the CA1 impaired long-term memory is not due to the alteration of locomotor activity and/or anxiety-like behaviors; we investigated whether SIRT6 overexpression affects locomotor activity and anxiety-like behaviors. Another two groups of rats (*n* = 10 per group) received LVSIRT6-GFP or LVGFP infusions in the CA1. Ten days later, all of the rats underwent the locomotor activity and elevated plus maze tests (Fig. 5A).

The behavioral data were analyzed using one-way ANOVA (LVSIRT6-GFP and LVGFP). This analysis revealed no significant effect of lentiviral type on the total distance (p > 0.05, Fig. 5B) or the time spent in the central area (p > 0.05, Fig. 5C) in the LA test. Similarly, no significant effect of lentiviral type on time spent in the open or closed arms (all p > 0.05, Fig. 5D) and on the entries into open or closed arms (all *p* > 0.05, Fig. 5E) in the elevated plus maze. These data indicate thatSIRT6 overexpression in the CA1 had no effect on locomotion or anxiety-like behaviors.

### Experiment 6: Intra-CA1 injection of LVSIRT6-GFP impaired the IGF-Akt signaling

**Figure 6:**
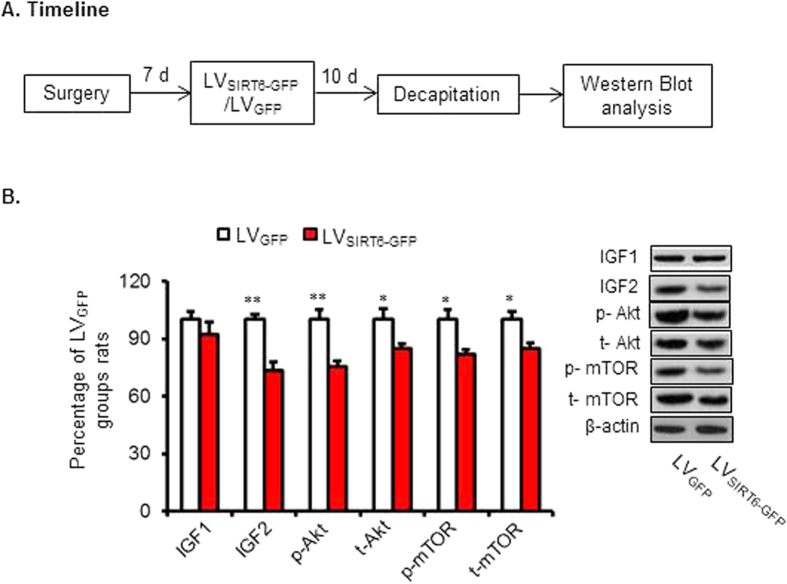
SIRT6 overexpression in the CA1 inhibited the activity of IGF signaling. (**A**) Timeline of the experiment. 10 days after LVSIRT6-GFP or LV_GFP_ infusions, all of the rats were decapitated for subsequent protein assessment. (**B**) Representative western blot showing decreased expression and phosphorylation of IGF signaling-related proteins after SIRT6 overexpression in the CA1. *p < 0.05, **p < 0.01, compared with LVGFP group.

IGF/Akt signaling has been reported to be inhibited by SIRT6 and hippocampal IGF/Akt signaling played a critical role in the formation of long-term memory^9^,^33^. We next tested whether effects of IGF-Akt signaling might underlie the impairment effects of LTM in mice withSIRT6 overexpression in the CA1 of the hippocampus. Two groups of rats (*n* = 5 per group) received LV_SIRT6-GFP_ or LV_GFP_ infusions into the CA1. Ten days later, the rats were decapitated for Western Blot analysis (Fig. 6A).

The Western blot data were analyzed using one-way ANOVA (LVSIRT6-GFP and LVGFP). This analysis revealed no significant effect of lentiviral type on the protein level of IGF1 (p > 0.05), but significant effects on the expression amounts of proteins (and/or phosphoprotiens) related to IGF signaling pathway, including IGF2 (100 ± 2.8%, 73.6 ± 4.4% separately, *F*_1,9_ = 26.025, *p* = 0.001, ES = 2.602), phosphorylated Akt (p-Akt) (100 ± 5.0%, 775.3 ± 2.8% separately, *F*_1,9_ = 18.505, *p* = 0.003, ES = 1.851), total Akt (t-Akt) (100 ± 5.6%, 84.6 ± 2.9% separately, *F*_1,9_ = 6.018, *p* = 0.040, ES = 0.602), phosphorylated mTOR (p-mTOR) (100 ± 5.0%, 81.8 ± 2.5% separately, *F*_1,9_ = 10.656, *p* = 0.011, ES = 1.066) and total mTOR (100 ± 4.4%, 84.9 ± 2.9% separately, *F*_1,9_ = 8.292, *p* = 0.021, ES = 0.830; Fig. 6B and Fig. S3). These data indicate that the impaired long-term memory induced by SIRT6 overexpression in the CA1 might be related to inhibitory activation of IGF/Akt signaling proteins.

## Discussion

In the present study, we evaluated the effect of SIRT6 overexpression in the hippocampal CA1 on the formation of contextual fear memory. We found that hippocampal CA1 protein level of SIRT6 was decreased after contextual fear conditioning. Microinjection of lentiviral SIRT6-GFP selectively increased the expression of SIRT6 without affecting other SIRTs (e.g. SIRT1 and SIRT2). SIRT6 overexpression in the CA1 selectively impaired the formation of long-term memory but not short-term memory. Furthermore, SIRT6 overexpression in the CA1 impaired the IGF/Akt signaling assessed by Western Blot analyses of amounts of proteins related to this pathway. This indicates that hippocampal CA1 SIRT6 overexpression impaired the formation of long-term fear memory, and this effect may be mediated by inhibition of the IGF/Akt signaling in the CA1 of the hippocampus.

Epigenetic regulation is critical for memory formation, which is dependent on alteration of synaptic-plasticity gene expression. Increasing evidence showed that histone modifications are powerful modulators of long-term potentiation (LTP) as well as memory formation^14^,^15^,^17^,^19^. Intrahippocampal injection of TSA, an HDAC inhibitor, immediately after learning produces enhancements in long-term memory without affecting short-term memory^49^. Hippocampal down regulation of HDAC3 is sufficient to enhance contextual fear memory, while HDAC3 in the nucleus accumbens (NAc) negatively regulated cocaine-context associated memory formation^50^,^51^. Although functions of HDACs have been well investigated, the functions of SIRTs in learning and memory are still not well understood. SIRT1 knockout mice exhibit a decrease in dendritic branching, branch length and complexity of neuronal dendritic arbors, and show altered hippocampal gene expression, which plays important roles in synaptic and structural functions. SIRT1 knockout mice or mutant mice lacking SIRT catalytic activity are associated with defects in synaptic plasticity in the hippocampus and induced cognitive deficits. Gao *et al.* reported that SIRT1 could positively modulate synaptic plasticity and memory formation via a microRNA-mediated mechanism^52^.

Formation of long term contextual fear memory requires transcriptional regulation of synaptic plasticity related genes in the hippocampus CA1 and a wide variety of transcripts are regulated by contextual fear conditioning^17^. In the present study, the result showed that both mRNA and protein level of SIRT6 in the CA1 was decreased 1.5 h after fear conditioning, which indicating the expression for SIRT6 may be in an activity-dependent manner. One mechanism to decrease the levels of SIRT6 is to increase its protein degradation; the other one is to attenuate its transcriptional expression. Recent studies have shown that SIRT6 expression could be regulated by C-FOS and some micro RNAs (miRNAs). Posttranscriptional regulation of SIRT6 by miR-33a, -33b, 34a and -766, which bind to the 3’ untranslated region of the SIRT6 transcript leading to decreased SIRT6 expression^53^,^54^. But the exact mechanisms by which SIRT6 gene expression is rapid decrease following fear conditioning are still elusive.

Furthermore, lentiviral-mediated overexpression of SIRT6 in the CA1 was used to evaluate the potential role of SIRT6 in memory formation. Behavioral results showed that SIRT6 overexpression in the CA1 significantly impaired the long-term but not short-term contextual fear memory (Fig. 3B). To identify whether overexpression of SIRT6 interferes with the formation of long-term contextual fear memory rather than its maintenance/expression, we added an experiment that LV-_SIRT6-GFP_ infusion was administered 24 h after the contextual fear memory test, and then we tested the contextual fear memory maintenance/expression. We found that LV infusion was administered 24 h after the contextual fear memory test had no effect on the contextual fear memory maintenance/expression in the test 2 (Fig. 4C). Together with the findings in the Fig. 3, it is clear that overexpression of SIRT6 in the hippocampal CA1 interferes with the formation of long-term contextual fear memory rather than its maintenance/expression. Moreover, we found that SIRT6 overexpression in the CA1 had no effect on locomotor activity and anxiety-like behaviors assessed by LA test and EPM test, indicating the negative effect of SIRT6 on memory formation was not due to the alteration of the locomotor activity or the emotional state of the rats.

SIRT6 deficiency was reported to induce hyperactivation of IGF-Akt signaling, which culminates in the development of cardiac hypertrophy and heart failure in mice. SIRT 6 could directly control insulin-like factor (IGF)/Akt at the level of chromatin through c-Jun and deacetylation of histone 3 at Lys 9 (H3K9)^33^. IGF2 as compared to the other family members, is more abundantly expressed in the adult brain and is found in region that are critically involved in memory consolidation, such as hippocampus and cortex. A recent study in rodent showed that hippocampal microinjection of recombinant IGF2 significantly enhances contextual memory formation and persistence^9^. Systemic treatment with IGF2 significantly enhances hippocampal- cortical-dependent memories, suggesting hippocampal IGF2 plays a critical role in brain plasticity and memory consolidation^55^. IGF2 mediated memory formation and enhancement also requires *de novo* protein synthesis. Many downstream signal molecules, such as mTOR and nucleus factor kappa B, have been reported to be critical for protein synthesis as well as memory formation (Refs regarding to AKT and mTOR). To further understand the potential mechanism underlying the effects of SIRT6 on contextual fear memory formation, amounts of proteins (and/or phosphoprotiens) related to IGF signaling were analyzed 10 days after Lentiviruses infection. Western Blot data showed that overexpression of SIRT6 in the CA1 induced significantly inhibited most of IGF signaling related molecules, providing that IGF signaling in the CA1 might mediate the regulatory effects of SIRT6 on long-term fear memory formation. Considering the role of SIRT6 in memory formation is poorly understood, more attention should be paid on the effects of SIRT6 on other learning/memory phenotypes (e.g. drug reward memory, working memory and so on). However, the conclusion of the causal role of SIRT6 in the regulation of IGF/Akt signaling during memory formation should be made with caution. Future studies should be carried out to verify whether SIRT6 directly target downstream members of the IGF/Akt signaling pathway during fear memory formation.

## Concluding remarks

In summary, our results indicate that the NAD^+^-dependent deacetylase, SIRT6 in the CA1 of the hippocampus negatively regulates the formation of long-term, but not short-term contextual fear memory. The negative effect of SIRT6 on memory formation was not attributable to alterations in the emotional state or locomotion activity of rats. Also, our work suggests that the inhibition of IGF/Akt signaling in the CA1 may be one of the mechanisms though which SIRT6 negatively regulate memory in hippocampus. Additional investigations should provide further insights into the unknown molecular mechanisms through which SIRT6 regulates learning and memory. The present study extends our understanding of the role of HDACs in learning and memory, highlighting its potential as a target for the treatment of neurodegeneration and conditions with impaired cognition, with implications for a wider range of CNS disorders.

## Additional Information

**How to cite this article**: Yin, X. *et al.* Overexpression of SIRT6 in the hippocampal CA1 impairs the formation of long-term contextual fear memory. *Sci. Rep.* **6**, 18982; doi: 10.1038/srep18982 (2016).

## Supplementary Material

Supplementary Information
