# Supplementary material for: Overexpression of SIRT6 in the hippocampal CA1 impairs the formation of long-term contextual fear memory
Source: Sci Rep. 2016 Jan 6;6:18982. doi: 10.1038/srep18982 (PMC4702175; doi:10.1038/srep18982)
Supplement: Supplementary Information [file srep18982-s1.pdf]

Overexpression of SIRT6 in the hippocampal CA1 impairs the formation of long-term contextual fear memory

Xi Yin[1#], Yuan Gao[2#], Hai-Shui Shi[2*], Li Song[2], Jie-Chao Wang[3], Juan Shao[4], Xu-Hong Geng[1], Gai Xue[5], Jian-Li Li[5], Yan-Ning Hou[5]

[1] Department of Functional region of Diagnosis, Hebei Medical University Fourth Hospital, Hebei Medical University, Shijiazhuang 050011, China;
[2]Department of Biochemistry and Molecular Biology, College of basic medicine, Hebei Medical University, Shijiazhuang 050017, China;
[3]Department of vasculocardiology, Hebei Province Geriatric Hospital, Shijiazhuang, 050011, China;
[4]Department of Senile Disease, the Third Hospital of Hebei Medical University, Shijiazhuang 050000, China;
[5]Department of Pharmacy, the Bethune International Peace Hospital of PLA, Shijiazhuang 050082, China;

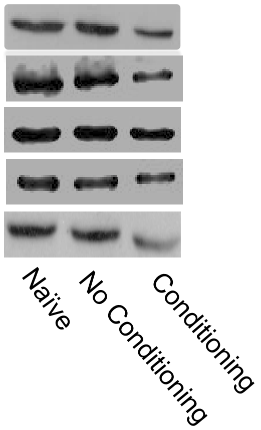

Fig. S1. Western blot results of SIRT6 expression in the CA1 region of rats 1h after fear conditioning.

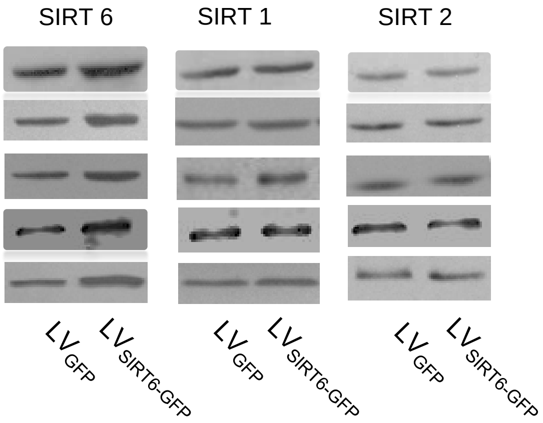

Fig. S2. Western blot results of several isoforms of SIRTs in the CA1 after $LV_{SIRT6\text{-}GFP}$ or LV_{GFP} infusion.

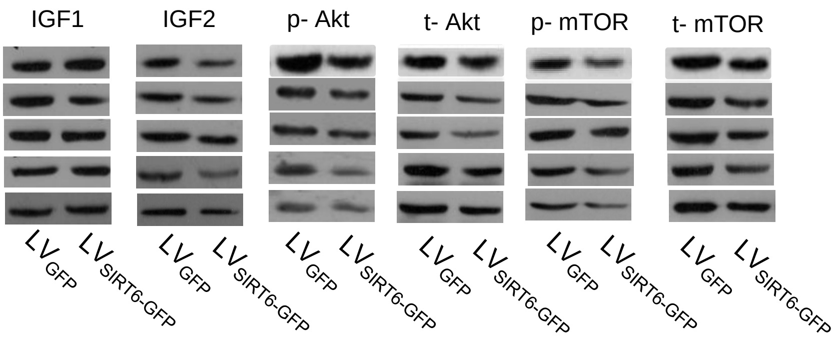

Fig.S3. Western blot results of expression and phosphorylation of IGF signaling-related proteins after SIRT6 overexpression in the CA1 of hippocampus.
